# Enhancing Innovation and Underlying Neural Mechanisms Via Cognitive Training in Healthy Older Adults

**DOI:** 10.3389/fnagi.2017.00314

**Published:** 2017-10-09

**Authors:** Sandra B. Chapman, Jeffrey S. Spence, Sina Aslan, Molly W. Keebler

**Affiliations:** ^1^Department of Behavioral and Brain Sciences, Center for BrainHealth, The University of Texas at Dallas, Dallas, TX, United States; ^2^Advance MRI, LLC, Frisco, TX, United States

**Keywords:** innovation, cognitive training, aging, creativity, CBF, functional connectivity, reasoning training, randomized trial

## Abstract

Non-invasive interventions, such as cognitive training (CT) and physical exercise, are gaining momentum as ways to augment both cognitive and brain function throughout life. One of the most fundamental yet little studied aspects of human cognition is innovative thinking, especially in older adults. In this study, we utilize a measure of innovative cognition that examines both the quantity and quality of abstracted interpretations. This randomized pilot trial in cognitively normal adults (56–75 years) compared the effect of cognitive reasoning training (SMART) on innovative cognition as measured by Multiple Interpretations Measure (MIM). We also examined brain changes in relation to MIM using two MRI-based measurement of arterial spin labeling (ASL) to measure cerebral blood flow (CBF) and functional connectivity MRI (fcMRI) to measure default mode and central executive network (CEN) synchrony at rest. Participants (*N* = 58) were randomized to the CT, physical exercise (physical training, PT) or control (CN) group where CT and PT groups received training for 3 h/week over 12 weeks. They were assessed at baseline-, mid- and post-training using innovative cognition and MRI measures. First, the CT group showed significant gains pre- to post-training on the innovation measure whereas the physical exercise and control groups failed to show significant gains. Next, the CT group showed increased CBF in medial orbitofrontal cortex (mOFC) and bilateral posterior cingulate cortex (PCC), two nodes within the Default Mode Network (DMN) compared to physical exercise and control groups. Last, significant correlations were found between innovation performance and connectivity of two major networks: CEN (positive correlation) and DMN (negative correlation). These results support the view that both the CEN and DMN are important for enhancement of innovative cognition. We propose that neural mechanisms in healthy older adults can be modified through reasoning training to better subserve enhanced innovative cognition.

## Introduction

Innovative cognition is widely recognized as a vital capacity, undergirding adaptive and flexible thinking. This cognitive domain is of interest in older adults due to its centrality to human cognition, intellect, decision-making, life achievement, resilience and psychological well-being (McFadden and Basting, 2010; Li et al., 2015; Beaty et al., 2016; Heilman, 2016; Palmiero et al., 2016; Saggar et al., 2016). Innovative thinking may be a pivotal cognitive capacity and brain function allowing one to respond effectively to challenging and constantly changing life demands (Saggar et al., 2016). Cognitive neuroscientists are becoming increasingly interested in elucidating the domain of innovative thinking, its neurobiological underpinnings; and whether this important capability can be enhanced (Fink et al., 2015). Thus, the present study offers one of the first pilot trials: (1) to examine whether innovative cognition can be improved as well as; and (2) to elucidate associated neural changes following cognitive or physical exercise training in healthy older adults.

Innovative thinking purportedly declines even before young adulthood (Kim, 2011) and may worsen with increasing age. Most aging evidence has focused largely on insidious cognitive declines in areas such as executive function, cognitive control and memory as well as losses in both structural and functional aspects of brain systems (Raz et al., 1997; Kennedy and Raz, 2009; Lu et al., 2011). Declines in these domains reportedly accumulate with increasing age even in the absence of frank dementia. The sparse evidence that does exist about age-related changes in innovative cognition is equivocal. Some evidence suggests that innovative thinking may follow the same degradation pattern as other executive functions and memory with a peak in early adulthood followed by accumulating declines starting as young as 30 s to 40 s (Alpaugh and Birren, 1977; McCrae et al., 1987; Reese et al., 2001). Other accounts have challenged this age-related loss pattern, showing preserved innovative cognition with aging (Roskos-Ewoldsen et al., 2008; Greenwood and Parasuraman, 2010). Li et al. (2015) have shown that real life success as reflected in publication productivity in university professors is related to maintaining innovative cognition with increasing age. Other researchers have shown that divergent thinking, one facet of innovative thinking, stabilizes in middle-age and is preserved across the lifespan (Palmiero, 2015) especially when controlling for processing speed (Elgamal et al., 2011).

With regard to age-related decline in brain function, significant changes occur in nodes across two brain networks linked to innovative thinking, namely, the central executive network (CEN) and the Default Mode Network (DMN; Beaty et al., 2016). Specifically, age-related declines are identified on measures of brain function including: (a) reductions in cerebral blood flow (CBF) as measured by arterial spin labeling MRI (ASL MRI) across brain regions (Lu et al., 2011); and (b) reduced functional connectivity in these specific regions (Sambataro et al., 2010; Hafkemeijer et al., 2012; Geerligs et al., 2015). With regard to how brain networks are linked to innovative thinking, the findings are inconsistent. Beaty et al. (2016) reports an inverse correlation between CEN and DMN that is associated with higher performance on innovation (Green et al., 2015; Beaty et al., 2016); whereas Takeuchi et al. (2012) reported increased connectivity between these regions in relation to innovation. Most participants in prior studies were college students. Therefore, it is unclear how the neural and cognitive findings generalize to healthy older adults or to older adults in response to training.

Whether or not innovative cognition can be improved in older adults remains an important issue to address. Clinical trials provide evidence that the neuroplasticity of the aging brain may indeed be harnessed to leverage a perspective shift towards one that refuses to accept the well-documented, insidious age-related loss as a definite outcome of the aging process (Chapman and Mudar, 2014; Rebok et al., 2014). Specifically, research findings reveal that a significant degree of age-related cognitive and brain losses can be halted, reversed or even inoculated against through the building of cognitive and brain reserves to stave off subsequent decline (Mahncke et al., 2006a; Anguera et al., 2013; Rebok et al., 2014; Chapman et al., 2015, 2016; Hohenfeld et al., 2017). Among a variety of opportunities to modify age-related losses, two non-pharmacological intervention-types have been shown to enhance cognition and neural systems, specifically cognitive training (CT) protocols (Levine et al., 2000; Mahncke et al., 2006b; Chapman and Mudar, 2014; Chapman et al., 2015, 2016) and physical exercise regimens (Kramer et al., 1999; Chapman et al., 2013, 2016). We previously reported that reasoning training (SMART^©^) improved performance on cognitive control measures of complex abstraction and working memory; whereas aerobic exercise improved immediate and delayed memory (Chapman and Mudar, 2014; Chapman et al., 2015, 2016). Linked to these cognitive gains, we also identified corresponding significant increases in resting CBF (Chapman et al., 2016). However, whether the cognitive (SMART^©^) protocol can improve innovative cognition and neural mechanisms has yet to be investigated in aging populations.

We extend our prior work by addressing whether the CT can also improve innovative cognition, influence brain systems and show correspondence between enhanced innovative cognition and brain changes in the same group of participants. The specific aims of this randomized pilot study were: (a) to determine whether innovative cognition in older adults can be improved through cognitive reasoning training; (b) to compare CBF changes following CT compared to physical training (PT) and wait-list controls; and (c) to elucidate brain mechanisms related to improved innovative thinking in cognitively normal adults (56–75 years of age). We set out to test three questions: would the CT affect: (1) innovative thinking as measured by the Multiple Interpretations Measure (MIM); (2) brain plasticity as measured by resting state CBF; and (3) correspondence between enhanced innovation performance and brain connectivity changes in two prominent brain networks, i.e., DMN and CEN.

## Materials and Methods

### Participants

A total of 140 subjects were screened in a multi-stage screening process comprising online, telephone and in-person questionnaires as well as a physical examination to ensure good health, see Supplementary Figure S1 for the consort chart. Participants were adults between the ages of 56 and 75 years, right-handed native English speakers, with at least a high school diploma, no history of psychiatric or neurological conditions, no history of medication changes or surgery entailing general anesthesia within 3 months, and no more than 20 min of aerobic activity, twice per week. The online questionnaire was followed up by a telephone interview to answer questions about the study, verify online responses, and screen for cognitive status using Telephone Interview for Cognitive Status-M ≥ 28. The third stage comprised of an in-person Intelligence Quotient (IQ) using Wechsler Abbreviated Scale of Intelligence (WASI) ≥ 80, mood screen using Beck Depression Inventory (BDI) ≤ 14 and cognitive status screen using Montreal Cognitive Assessment (MoCA) ≥ 26. Finally, in the fourth stage, a physician examined each participant’s physical ability to comply with the study’s exercise requirements through an in-person physical assessment of height, weight, waist circumference, Body Mass Index (BMI) < 40, hypertension screen, basic blood test and graded stress test. Specifically, participants underwent a maximal oxygen consumption (relative VO_2_ max: mL/kg/min) exercise stress test to assess maximal exercise capacity as well as blood pressure/ECG responses and rating of perceived exertion (RPE) according to the Borg scale, range: 6–20 (Borg, 1990). A repeat of this rigorous assessment was carried out at all three time points during and following the training.

This study was carried out in accordance with the recommendations of Institutional Review Boards (IRB) of University of Texas Southwestern Medical Center, University of Texas at Dallas and The Cooper Institute. All subjects gave written informed consent in accordance with the Declaration of Helsinki. The participants were then randomized using a block randomization schedule stratified by gender into one of three groups: (CT, *n* = 19), (PT, *n* = 19) and Wait-listed control (CN, *n* = 20). All participants in the PT and CT groups were required to complete at least 90% of the training sessions over the 3-month period. No significant differences in age, gender, estimated IQ, MoCA, Telephone Interview of Cognitive Status-Modified (TICS-M) were noted between groups (*p* > 0.05), as shown in Table 1. This study was registered at Clinical Trials.gov, NCT#00977418.

**Table 1:** Baseline subject characteristics and total number of subjects per group, assessments and MRI technique (mean ± SD).

	Control	Physical training	Cognitive training	Range	*p*-value
Age	64.0 ± 3.6	64.0 ± 4.3	61.8 ± 3.3	56–75	0.50
Gender (M/F)	5/15	6/13	8/11	−	0.45
IQ	120.9 ± 10.5	117.5 ± 9.9	121.6 ± 8.0	88–136	0.45
MoCA	28.2 ± 1.4	27.8 ± 1.5	27.9 ± 1.4	25–30	0.72
TICS-M	29.6 ± 2.0	30.7 ± 2.0	29.4 ± 2.2	27–36	0.14
BDI	5.5 ± 4.7	3.0 ± 2.8	3.3 ± 2.4	0–14	0.07
BMI	26.4 ± 3.3	27.7 ± 4.5	25.8 ± 3.6	19–38	0.34
VO_2_ Max	19.9 ± 4.0	19.3 ± 3.3	20.8 ± 5.0	13–30	0.52
Participants (*n*)					
MIM cognitive testing	20	19	19		
pCASL MRI	18	18	13		
fcMRI	16	15	15		

*Two-sample t-test was conducted to assess potential baseline differences. IQ, Intelligence Quotient; MoCA, Montreal Cognitive Assessment; TICS-M, Telephone Interview of Cognitive Status-Modified; BDI, Beckman Depression Inventory; BMI, Body Mass Index; VO_2_ Max, maximal oxygen consumption; MIM, Multiple Interpretations Measure to measure innovation; pCASL, pseudo-Continuous Arterial Spin Labeling; fcMRI, functional connectivity MRI*.

### Cognitive Training Program

The CT program used in this study is an evidence-based, manualized program focused on enhancing top-down executive functioning: Strategic Memory Advanced Reasoning Training or SMART^©^ (Chapman and Mudar, 2014; Chapman et al., 2015, 2016). For treatment fidelity, the sessions for all participants in the CT group were led by the same clinician, whose three-stage training process included reviewing literature on the program, observing other trained clinicians and leading non-study SMART^©^ training groups under the supervision of a trained clinician. The SMART^©^ training sessions were comprised of 12 1-h in-person small group (*n* ≤ 5) sessions held once a week for 12 weeks. Additionally, each participant was assigned two 1-h pencil and paper assignments to complete at home each week for a total of 24 h of solo work, for a total of 36 h over the course of the study. In addition to completing the independent assignments, each participant kept a log of the assignments, which included the total amount of time spent and task completion.

SMART^©^ trained and provided practice of three metacognitive strategies for each of the complex cognitive functions of Strategic Attention, Integrated Reasoning and Innovation. As stated in Chapman et al. (2016), Distinct Benefits of Cognitive vs. PT, *Strategic Attention* is the ability to filter important information from less relevant data which is routinely necessary in life to efficiently manage time and cognitive resources by prioritizing daily goal setting, blocking distractions, intentionally single tasking, and scheduling regular mental breaks during the day. *Integrated Reasoning* teaches individuals to synthesize information at deeper levels of interpretation by abstracting the essence or extracting key goals for tasks. Strategies for Integrated Reasoning exert cognitive control to “zoom in” on the important details or steps to a goal, then rapidly “zooming out” to synthesize, and abstract big picture ideas/goals, followed by “zooming deep and wide” to construct generalized application of derived ideas, interpretations, or goals-completed. It is a skill that allows one to make informed decisions and solve problems in dynamic and demanding environments. The strategies of Innovation encourage fluid and flexible thinking, perspective taking and problem solving. *Innovation* focuses on flexibly updating ideas and perspectives and continually seeking ways to improve everyday tasks. These three core strategies were trained in the first 3 weeks of in-person group meetings so that participants could understand the basics of SMART^©^. The remainder of the training hours, participants practiced generating synthesized ideas and relevant application of the strategies to everyday life. Trainees received feedback from the trainer not only relative to performance on in-session group interactions regarding complex cognitive activities but also regarding their responses to applied activities. SMART trains individuals to approach challenging cognitive tasks with a brain prepared to think deeply, to continually synthesize data encountered daily (e.g., movies, medical information, speeches) and to practice innovative thinking by generating diverse interpretations, solutions and perspectives.

### Physical Training Program

The PT program used in the study, similar to the CT program, was comprised of three 1-h exercise sessions per week for 12 weeks. Every exercise session of aerobic activity occurred under supervision of trained personnel, an exercise physiologist and a nurse practitioner, with alternate use of treadmill walking and stationary cycling. By monitoring participants every 5 min, the supervising trainers ensured that they maintained 50%–75% of their VO_2_ max during the individual sessions. Sessions were structured to include 5-min warm-up and cool-down periods with specified slower speeds and 50 min at the rate necessary to maintain the required VO_2_ max For a complete description of both training protocols employed, interested readers are encouraged to reference “Distinct brain and behavioral benefits of cognitive vs. PT: a randomized trial in aging adults” (Chapman et al., 2013).

### Multiple Interpretations Measure (MIM)

A shortcoming of assessment batteries for innovative cognition is the limited ability to measure novelty and relevance of ideas in responses that typify naturally occurring cognitive activities and challenges. Commonly used measures to characterize innovation include a variety of divergent thinking tasks such as Guilford’s Alternative Use Task (e.g., list as many different uses of cardboard boxes, a brick, pencil, etc.), other verbal fluency tasks (e.g., list as many words as possible that begin with the letter “d” or exclude the letter “k”), ideational fluency like some of the prompts present in the Torrance Test of Creative Thinking (e.g., ask as many questions as possible regarding a provided image or object, list as many consequences as possible for a given image, list as many improvements as possible for a toy, and as many consequences as possible for impossible scenarios like people no longer needing sleep; Guilford, 1967; Wallach, 1968; Kaufman and Sternberg, 2006; Runco and Acar, 2012; Runco and Jaeger, 2012). Whereas these measures may be informative, these are less common cognitive challenges faced in everyday life in older adults and may lack ecological validity.

For the present study, we utilized The MIM, a subtest of Test of Strategic Learning (TOSL). This test is comprised of three expository texts about an historical person who is unknown but has generalizable life experiences from which distinct high-level themes may be gleaned, e.g., the meaning of success, self-actualization, courage, strength during moments of adversity, etc. One of the three versions was randomly administered at each assessment time point: pre- (T1), mid- (T2) and post-training (T3) periods. The primary scale of the Test of Strategic Learning measures cognitive control of complex abstraction as represented through the ability to understand and synthesize the overall meaning in a synopsis of text, much like you would in the abstract of an article or a synopsis of a movie. The Multiple Interpretation Measure subtest measures an individual’s ability to generate multiple interpretations of the expository text, a task motivated by the work of Kaufman and Sternberg (2006). Specifically, participants are asked to construct as many high-level interpretations as possible that can be drawn from the expository texts but which are not explicitly stated.

In this way, the MIM subtest taps the ability to combine presented information with their world knowledge in a multitude of ways. This subtest that solicits multiple interpretations represents a real-life task, similar to what a person could encounter in everyday life when they express a range of ideas and/or solutions. For instance, when engaged in conversation, there are an infinite number of possible interpretations for a movie, political speech, medical scenario dilemma, or future financial advice. These interpretations are self-generated ideas, which are not explicitly conveyed in the texts. Instead, individuals use cognitive control processes to create abstracted responses. Abstracted interpretations require the individual to decipher meanings expressed in the immediate context and combine these meanings with their own experiences and world knowledge to construct plausible and relevant responses.

For scoring purposes, every interpretation was first rated along two dimensions: either high-quality (HQ) or Other-type. Responses were coded as HQ when they were judged to convey generalized/abstracted ideas that showed an ability to combine the meanings from the text within the context of more generalized real world knowledge. In short, HQ responses were those that represented a depth of understanding and synthesis of meaning whereas Other-type responses tended to represent more of a reiteration of literal facts or obvious ideas from the text. To exemplify, one of the texts of the MIM describes the life of a man who was not considered a success during his lifetime in terms of predominant societal measures, who nonetheless in retrospect made incredible contributions to humankind. A specific example of a HQ interpretation is, “Often the perspective of time can redeem a person’s ideas and ideals”. Or “Empathy can impact the lives of many by creating societal change”. Example of Other-type responses would be “He had a lot of jobs and failed at them all” or “He never seemed to be satisfied with his choices”. The first examples clearly represent synthesized statements that generalize beyond what is explicitly stated in the text whereas the latter responses relate only to the meaning as conveyed in the text.

Three clinicians, trained in the scoring method for the measure, utilized a coding manual with sample responses to make response judgments. Three raters scored the responses separately and were blinded to the participant’s group membership and time interval of test, i.e., whether they were scoring T1, T2 or T3. Disagreements on scores were resolved by consensus. Changes in participants’ innovative responses over time were determined by comparing the number of HQ interpretations by training group (i.e., T1 to T2 and/or T3).

### MRI Experiment

MRI investigations were performed on a 3 Tesla MR system (Philips Medical System, Best, Netherlands). A body coil was used for radiofrequency (RF) transmission and an 8-channel head coil with parallel imaging capability was used for signal reception. We used different MRI techniques to investigate changes at rest: a pseudo-Continuous Arterial Spin Labeling (pCASL) sequence was used to measure CBF, functional connectivity MRI (fcMRI) was used to assess functional connectivity of the brain. Additionally, a high-resolution *T*_1_-weighted image was acquired as an anatomical reference. The details of imaging parameters and their processing techniques are provided below.

Imaging parameters for pCASL experiments were: single-shot gradient-echo EPI, field-of-view (FOV) = 240 × 240, matrix = 80 × 80, voxel size = 3 × 3 mm^2^, 27 slices acquired in ascending order, slice thickness = 5 mm, no gap between slices, labeling duration = 1650 ms, post-labeling delay = 1525 ms, time interval between consecutive slice acquisitions = 35.5 ms, TR/TE = 4020/14 ms, SENSE factor 2.5, number of controls/labels = 30 pairs, RF duration = 0.5 ms, pause between RF pulses = 0.5 ms, labeling pulse flip angle = 18°, bandwidth = 2.7 kHz, echo train length = 35, and scan duration 4.5 min. The sequence parameters for fcMRI were FOV = 220 × 220, matrix = 64 × 64, slice thickness = 4 mm, no gap between slices, voxel size = 3.44 × 3.44 × 4 mm^3^, 36 axial slices, TR/TE = 2000/30 ms, flip angle = 70°, 120 image volumes, and scan duration = 4 min. The high-resolution *T*_1_-weighted image parameters were magnetization prepared rapid acquisition of gradient-echo (MPRAGE) sequence, TR/TE = 8.3/3.8 ms, shot interval = 2100 ms, inversion time = 1100 ms, flip angle = 12°, 160 sagittal slices, voxel size = 1 × 1 × 1 mm^3^, FOV = 256 × 256 × 160 mm^3^, and duration 4 min.

pCASL image series were realigned to the first volume for motion correction (SPM5’s realign function, University College London, UK). An in-house MATLAB (Mathworks, Natick, MA, USA) program was used to calculate the difference between averaged control and label images. Then, the difference image was corrected for imaging slice delay time to yield CBF-weight image, which was normalized to the Brain template from Montreal Neurological Institute (MNI). This procedure was carried out using a nonlinear elastic registration algorithm, Hierarchical Attribute Matching Mechanism for Elastic Registration (HAMMER, University of Pennsylvania, PA, USA). The HAMMER algorithm detects and corrects for region-specific brain atrophy which is commonly seen in elderly subjects. Last, the absolute CBF was estimated by using Alsop and Detre’s equation in the units of mL blood/min/100 g of brain tissue (Aslan et al., 2010).

For voxel-based analyses (VBA), the individual CBF maps were spatially smoothed (with full-width half-maximum (FWHM) of 4 mm) to account for small differences in sulci/gyri location across subjects. For cluster extent inference, we used *3dClustsim* in AFNI (NIMH Scientific and Statistical Computing Core, Bethesda, MD, USA), which controls false-positive activation clusters over the set of all activation clusters throughout the whole-brain volume. We refer to this procedure in the “Results” Section as family-wise error correction (FWE corrected). For cluster inference, we tested the volume of clusters, which is conditional on two criteria: smoothness of the voxel map and cluster-defining threshold. We estimated the smoothness to be 9.3 mm FWHM (inherent smoothness plus additional smoothness applied—described above) and set the cluster-defining threshold to the 99.5th percentile of *t*-statistic distribution. Then, the minimum cluster size of 98 voxels (784 mm^3^) yielded an FWE-corrected significance level of 0.05.

Functional connectivity images were analyzed by using AFNI (NIMH Scientific and Statistical Computing Core, Bethesda, MD, USA). The dataset was preprocessed with slice timing correction, motion correction (realignment), removal of the linear trend, smoothing by a Gaussian filter with a FWHM of 6 mm and band-pass filtering (0.01–0.1 Hz) to keep appropriate frequency fluctuations. Next, images were spatially normalized to MNI template. In the DMN analysis, we correlated the time series of the orbitofrontal cortex (OFC) and posterior cingulate cortex (PCC) regions (an ROI analysis since the regions were significant in the CBF analysis and are part of DMN). Then, a Pearson correlation was conducted between the time series of PCC and OFC; followed by a z-transformed using Fisher’s transformation. In the CEN analysis of functional connectivity, the preprocessed images were analyzed using a seed-based approach by choosing bilateral dorsolateral prefrontal [± 45 + 16 + 45] cortices based on MNI coordinates (Chapman et al., 2015). The cross-correlation coefficient between these seed voxels and all other voxels was calculated to generate a correlation map. Next, the correlation maps were converted to a z-transformed using Fisher’s transformation. Last, an ROI analysis was performed based on two CEN regions: dorsolateral prefrontal cortex (DLPFC; composed of BA 9 and 46) and inferior parietal cortex (IPC). The functional ROIs were defined as follows: first, each region’s anatomical region was defined based on Talairach Daemon database in AFNI. Then, a functional ROI was defined by choosing the top 200 voxels at each time point (i.e., T1, T2 and T3) and the intersection (i.e., common voxels) of the masks was calculated (Chapman et al., 2015). The CEN Z-Score was calculated by averaging the values of the all four nodes of CEN: L/R DLPFC and L/R IPC.

### Statistical Analyses

All tests were *t*-statistic contrasts of parameter estimates from a linear mixed model. The computations were implemented in the R computing language^1^. We modeled HQ innovations as additive effects of training type (CT, CN, control and PT), time of assessment (T1—baseline, T2—mid-training and T3—post-training), and the interaction between type of training and assessment period in a standard linear mixed effects model framework. Two variance components—one due to variability across subjects, and one due to variability in the same subject over time—were included to account for the different levels of variability and estimated by restricted maximum likelihood. We were primarily interested in how the groups differed across the training sessions. Thus, we hypothesized that the CT group would show a larger positive change in mean number of HQ innovations by T2 and/or T3, relative to the control and PT groups. This hypothesis led to the following one-sided *t-statistic* contrasts of means from the linear mixed effects model: (1) time contrasts (T23 − T1) for each group, where we define (T23 − T1) = (T2 + T3)/2 − T1 as the “sustained change” following training; (2) interaction contrasts (T23 − T1)_CT_ − (T23 − T1)_CN_ and (T23 − T1)_CT_ − (T23 − T1)_PT_. One additional interaction contrast was also tested as (T23 − T1)_CT_ − (T23 − T1)_CN/PT_, where CN/PT denotes the average of the two control groups. These six contrasts were tested as single degree-of-freedom *t-tests* from the linear mixed effects model without multiple comparisons adjustments.

We modeled CBF similarly. That is, in the VBA, we used the same linear mixed effects model for voxel-level CBF as noted above: training type (CT, CN, PT), assessment period (T1, T2, T3), and their interaction. Our hypothesis for CBF was also similar to that of HQ innovations—we hypothesized that the CT group would show a larger positive change in mean CBF by T2 and/or T3, relative to the control and PT groups. For this hypothesis, we tested only the single interaction contrast (T23 − T1)_CT_ − (T23 − T1)_CN/PT._ We did not, however, hypothesize specific regions of the brain in which we expected this CBF relationship. Therefore, as noted above in the description of our VBA analysis, we FWE corrected through AFNI’s cluster-extent inference.

Last, we used separate linear models to assess the HQ innovations/DMN connectivity relationship and the HQ innovations/CEN connectivity relationship. In the first linear model the dependent variable was (T23 − T1) for HQ innovations, and the independent variable was (T23 − T1) for DMN connectivity on a z-transformed scale (described above). In the second linear model, based on the CEN findings of Chapman et al. (2015), the independent variable was “transient change” (T2 − T1) for CEN connectivity on a z-scale, and the dependent variable was, similarly, (T2 − T1) for HQ innovations. For both models, training type (CT/CN/PT) was also included as an additive term, as well as the interaction of training type with the independent variable DMN or CEN connectivity change, respectively. From these models, we calculated regression coefficients for each group and tested their respective differences from zero as *t*-statistics. Additionally, we tested the single degree-of-freedom interaction contrast B_CT_ − B_CN/PT_, where B denotes the estimated regression coefficient. In the first model our primary hypothesis was that the functional relationship would be restricted to the CT group relative to controls and the PT group, yielding a significant interaction test, but without a directional hypothesis. In the second model, however, our hypothesis was that the HQ innovations/CEN connectivity relationship was positive and restricted to the CT group. This directional hypothesis was also based on the CEN findings of Chapman et al. (2015).

## Results

All control (CN, *n* = 20), physical training (PT, *n* = 19) and cognitive training (CT, *n* = 19) participants completed the neurocognitive assessments at each time point. However, several participants in the control (CN), CT and physical exercise (PT) groups were not included in the analysis due to incomplete MRI time points, gross movement of >3 mm, and >3° and/or artifacts. All physical exercise and CT participants were required to complete at least 90% of training sessions over the 3-month training period, which means they completed 32 h or more of the 36 h of training. One baseline-only measurement for the neurocognitive assessment of one CT participant was removed because it had been scored incorrectly. No participant was excluded due to missing too many sessions to meet the 90% criterion.

### Neurocognitive Analysis

**Figure 1:**
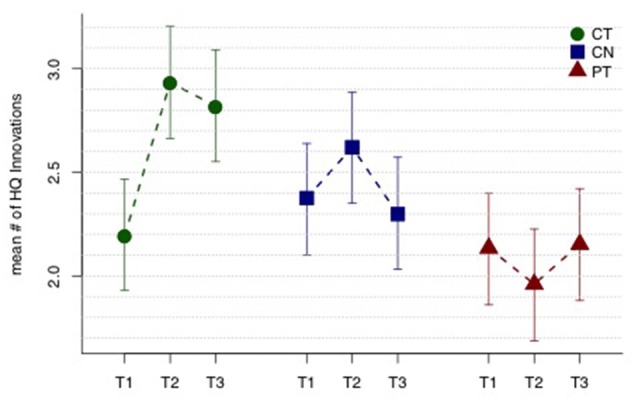
High-quality (HQ) innovation results. Mean number of HQ innovations across three assessment periods T1, T2 and T3 (baseline, mid-training, and post-training, respectively). The cognitive training (CT) group shows a sustained improvement in mean HQ innovations (T23 − T1), while the controls (CN) and physical training (PT) groups do not. See text and Table 2 for tests of the relevant contrasts. Error bars indicate 95% least significant intervals for contrasts T23 − T1 by group.

Figure 1 displays mean HQ innovations for each group and each assessment period, and Table 2 lists all the relevant contrasts of interest from the linear mixed model. The CT group shows a significant mean “sustained increase” in number of HQ innovations from T1 to T23 (*t*_109_ = 2.23, *p* = 0.014); whereas the same contrast for the control and PT groups were not significant (*t*_109_ = 0.44, *p* = 0.33 and *t*_109_ = −0.08, *p* = 0.53, respectively). Comparing the sustained increase for the CT group with the comparable change in the control group and the PT group (i.e., from T1 to T23), we found that the sustained increase for the CT group is marginally larger than the control group (*t*_109_ = 1.30, *p* = 0.098) and, similarly, marginally larger than the PT group (*t*_109_ = 1.63, *p* = 0.053). Averaging the sustained change over the two control groups (i.e., CN and PT), we find that the sustained increase for the CT group is also marginally larger than the average change of the controls and PT groups (*t*_109_ = 1.69, *p* = 0.047).

**Table 2:** HQ innovation results.

Mean time contrasts	Estimate	SE	*t*-statistic	*df*	*p*-value
(T23 − T1)_CT_	0.739	0.331	2.23	109	0.014
(T23 − T1)_CN_	0.139	0.316	0.44	109	0.33
(T23 − T1)_PT_	−0.024	0.300	−0.08	109	0.53
**Mean interaction contrasts**
(T23 − T1)_CT_ − (T23 − T1)_CN_	0.6	0.462	1.3	109	0.098
(T23 − T1)_CT_ − (T23 − T1)_PT_	0.763	0.468	1.63	109	0.053
(T23 − T1)_CT_ − (T23 − T1)_CN/PT_	0.681	0.403	1.69	109	0.047

*Contrasts from the linear mixed model for HQ innovations for cognitive training (CT), control (CN) and physical exercise (PT) groups*.

### CBF Analysis

**Figure 2:**
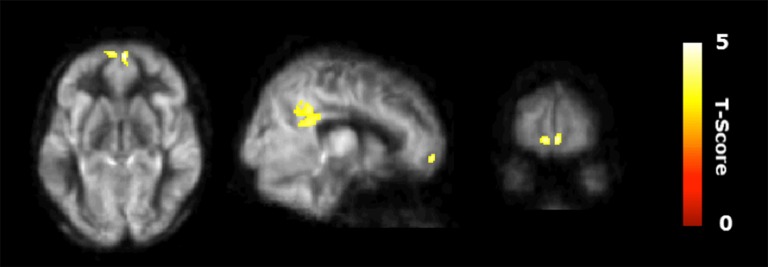
Regional cerebral blood flow (CBF) results. Voxel-based analysis for the interaction contrast described in text, superimposed on an average CBF map of all participants. Both cluster volumes *k* = 3792 mm^3^ for posterior cingulate cortex (PCC) and *k* = 992 mm^3^ for medial orbitofrontal cortex (mOFC) are significant at an family-wise error correction (FWE) alpha level of 0.05 (*k* = 784 mm^3^).

Figure 2 shows the results of the interaction contrast described above for the VBA of the CBF maps. That is, we tested whether the sustained increase for the CT was greater than that of the average change between the control (CN) and physical exercise (PT) groups. A significantly larger increase in blood flow was observed at T23 in bilateral medial orbital frontal cortex (mOFC) and bilateral PCC of the CT group compared to the PT/control group, shown in Figure 2. Both mOFC and PCC are major nodes of DMN (Fox et al., 2005). Table 3 summarizes these findings for cluster-level inference as well as descriptive statistics for peak voxel within cluster. Cluster volumes larger than 784 mm^3^ (FWE alpha level of 0.05) yield FWE *p*-values less than 0.05. Our observed cluster volumes for PCC and mOFC are 3792 and 992 mm^3^, respectively.

**Table 3:** Regional CBF results.

			MNI		
Brain regions	BA	Cluster size (mm^3^)	*X*	*Y*	*Z*	*T*-Value
CT > CN/PT						
L/R posterior cingulate cortex	31/23	3792	−4	−44	36	4.76
L/R medial orbitofrontal cortex	11/10	992	6	64	−8	4.91

*Regions that showed significant cerebral blood flow (CBF) increase at rest in cognitive training (CT) compared to control (CN) and physical training (PT) groups. The coordinates depict the peak of clusters*.

### Neurocognitive and Regional Connectivity Relationship

**Figure 3:**
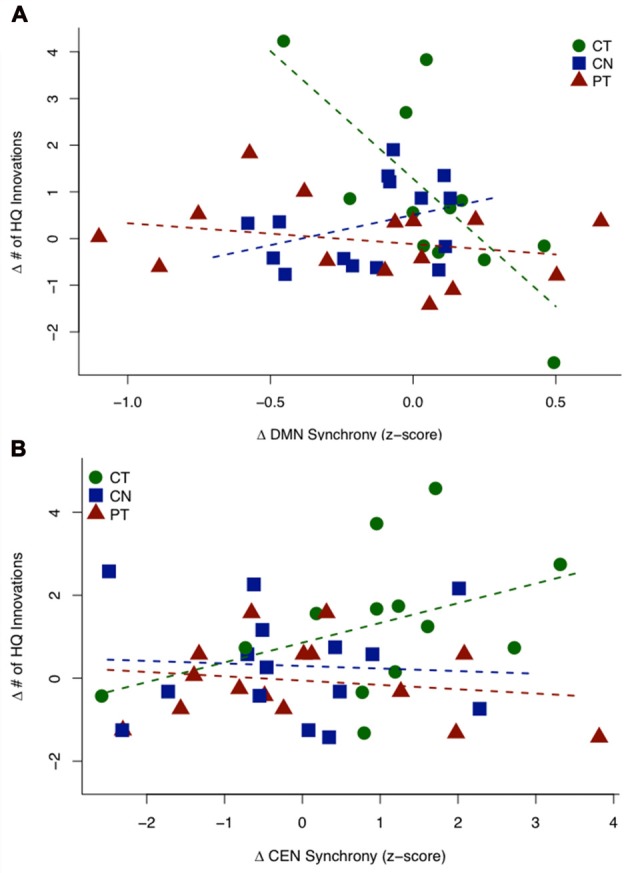
HQ innovation changes in relation to changes in connectivity of default mode network (DMN) and central executive network (CEN). **(A)** Scatterplot of the sustained change (T23 − T1) in HQ innovation scores against the sustained change (T23 − T1) in DMN connectivity z-scores. The CT group shows a significant negative relationship, while the controls (CN) and PT groups do not show significant relationships between behavior and connectivity. **(B)** Scatterplot of transient change (T2 − T1) in HQ innovation scores against the transient change (T2 − T1) in CEN connectivity z-scores (outlier removed, see text). The CT group shows a significant positive association, while the controls (CN) and PT groups show no significant relationship. Table 4 displays the regression statistics from both linear models.

Figure 3A shows a scatterplot of the relationship between the sustained change in functional connectivity of the DMN and the sustained change in High Quality innovation scores, coded separately for each group. Table 4 displays the regression statistics from the linear model. An inverse relationship was found for the CT group (*t*_36_ = −4.57, *p* < 0.001); whereas the control (CN) and physical exercise (PT) groups showed no significant relationship (CN: *t*_36_ = 1.15, *p* = 0.25; PT: *t*_36_ = −0.81, *p* = 0.43). Furthermore, the inverse relationship for the CT group was significant relative to the control (CN) and physical exercise (PT) groups (interaction test in Table 4: *t*_36_ = −4.36, *p* < 0.001).

**Table 4:** HQ innovation changes in relation to changes in connectivity of default mode network (DMN) and central executive network (CEN).

A	∆ HQ innovation as a function of ∆ DMN connectivity				
	Group-specific coefficient	Estimate	*t*-statistic	*df*	*p*-value
	B_CT_	−5.46	−4.57	36	<0.001
	B_CN_	1.31	1.15	36	0.257
	B_PT_	−0.45	−0.81	36	0.425
	**Interaction contrast**	**Estimate**	***t*-statistic**	***df***	***p*-value**
	B_CT_ − B_CN/PT_	−5.89	−4.36	36	<0.001
**B**	**∆ HQ innovation as a function of ∆ CEN connectivity**				
	**Group-specific coefficient**	**Estimate**	***t*-statistic**	***df***	***p*-value**
	B*_CT_	0.476	1.837	37	0.037
	B_CN_	−0.061	−0.239	37	0.594
	B_PT_	−0.104	−0.483	37	0.684
	**Interaction contrast**	**Estimate**	***t*-statistic**	***df***	***p*-value**
	B*_CT_ − B_CN/PT_	0.559	1.81	37	0.039

Regression statistics from linear model for ∆HQ innovation as a function of (A) ∆ DMN connectivity and (B) ∆ CEN connectivity (*outlier removed, studentized residual = −4.16, Bonferroni p-value = 0.010) for the cognitive training (CT), control (CN) and physical training (PT) groups.

Figure 3B shows a scatterplot of the relationship between the transient change in functional connectivity of the CEN and the transient change in HQ innovation scores, coded separately for each group. One subject in the CT group has been removed based on outlier diagnostics (studentized residual = −4.16; outlier test—Bonferroni *p*-value = 0.010), see Supplementary Figure S2. A positive association was found for the CT group (*t*_37_ = 1.837, *p* = 0.037); whereas the control (CN) and physical exercise (PT) groups showed no significant relationship (CN: *t*_37_ = −0.239, *p* = 0.594; PT: *t*_37_ = −0.483, *p* = 0.684). Furthermore, the positive relationship for the CT group was significant relative to the control (CN) and physical exercise (PT) groups (interaction test in Table 4: *t*_37_ = 1.81, *p* = 0.039). In Table 4, we display the regression statistics from the linear model for ∆HQ innovation as a function of ∆DMN and ∆CEN, respectively.

## Discussion

This randomized pilot study evaluated whether innovative cognition was improved in a group of older adults (56–75 years) in response to CT vs. physical exercise training (PT) or a wait-list control group (CN). In previously published research, we showed that CT improved cognitive control on measures of complex abstraction and working memory; whereas physical exercise enhanced immediate and delayed memory (Chapman and Mudar, 2014; Chapman et al., 2015, 2016). In the current article, we used the same cohorts, but compared a distinct measure from that previously reported, to examine this new question as to whether the CT protocol would also improve innovative cognition. The outcome measure was a novel cognitive innovation task intrinsically related to real life demands, i.e., being able to formulate multiple interpretations for a lengthy expository text.

Our preliminary results can be summarized as three key findings. First, we found that the CT group showed significant gains in high quality innovation performance. In contrast, neither the exercise nor the control group showed significant changes in innovation performance over time. Second, we identified mechanisms related to training-induced brain changes, namely increases in CBF within the CT group only. The CT group showed significant change from baseline bilaterally in the mOFC and the PCC, major nodes in the DMN. Lastly, we found significant associations between changes in high quality Innovation scores and the connectivity of two major neural networks, the CEN and the DMN using resting state fcMRI in the CT group. Specifically, individuals in the CT group with high quality innovation scores showed increased connectivity in CEN nodes (a positive correlation) as contrasted with decreased connectivity in DMN nodes (a negative correlation) on resting state fcMRI.

Overall, the findings support a potential to harness latent innovative thinking capacity and neuroplasticity in a cognitively normal older adult population (56–75 years) with a short-term cognitive reasoning training protocol, namely SMART^©^. These results add to growing data showing that older adults benefit from different forms of CTs (Mahncke et al., 2006b; Ball et al., 2010; Greenwood and Parasuraman, 2010; Anguera et al., 2013; Hohenfeld et al., 2017). The current study is one of the first known studies to show gains in innovative cognition and corresponding neural networks linked to reasoning training in older adults. Taken together with prior research showing enhanced neurocognitive effects with reasoning training (Chapman et al., 2013, 2016), the present findings support the potential for such training to have broad-based benefits manifested not only on measures of cognitive control but now these results also implicate a potential to improve innovative cognition in middle-age to older adults. This promise of improved innovative cognition capacity in cognitively normal adults warrants further validation in a larger study.

Our evidence suggests that the CT (SMART^©^) may be deployed to induce an experience-driven neuroplasticity in cognitively normal older adults. This enhanced innovative cognition performance had a direct association with gains in the CEN regions’ connectivity but an inverse association with the DMN regions’ connectivity in the CT group. The advantageous patterns of connectivity within the CEN and DMN are reinforced by previous evidence linking such a dynamic relation to innovative cognition (Greicius et al., 2003; Beaty et al., 2016). Further evidence that the neural changes reflect positive brain reorganization with CT is supported by the distinct pattern for the CT group only with no significant innovation or neural changes for the physical exercise (PT) and control (CN) groups. Thus, we propose that the change in connectivity of CEN and DMN following reasoning training may represent a redesigned “healthier neural mechanism” in older adults that is able to better subserve enhanced innovative cognition. Specifically, continued research toward this effort would help determine if reasoning training builds a more resilient system to counteract failure between two major networks; which previously has been linked to inefficient cognitive performance in healthy and compromised brains (Bonnelle et al., 2011, 2012). This interacting neural pattern between networks is consistent with the claim by Beaty et al. (2016) that innovative thinking engages dynamic interactions of large-scale brain networks, especially the CEN and DMN.

The nature of this complex and dynamic interaction of the CEN and DMN in relation to innovative thinking is equivocal. Jung et al. (2013), concluded in their review article that both increased and decreased brain “fidelity” across major brain networks was linked to creative innovation. In contrast, other studies report the opposite inverse innovation-connectivity relation between the two networks in relation to elevated divergent thinking performance, an aspect of innovative cognition (Takeuchi et al., 2012; Benedek et al., 2014; Mayseless et al., 2015; Beaty et al., 2016). Despite the disparity in directionality of CEN and DMN in support of innovative cognition, the consensus supports a dynamic interplay between the two functionally distinct but complementary networks (Jung et al., 2013). We propose that the complex operations of innovative thinking are not isolated to single neural hubs, but rather are supported through the involvement of at least two brain networks of DMN and CEN.

A number of factors may contribute to this seemingly disparate pattern across studies such as: (1) the nature of the innovative paradigm; (2) resting-state vs. task-induced studies; (3) age of participants; and (4) single time point measurement vs. longitudinal measurement in response to an intervention. First, the measure of innovation that we used in the present study is distinct from those used in prior work. Our innovative task taps top-down processes, drawing upon controlled retrieval of information, combining and integrating the selected ideas with world knowledge to generate and create a multitude of abstract interpretations. Second, different mechanisms are tested when comparing resting-state vs. task-induced brain imaging. The majority of studies that have shown increased DMN with higher creativity were task-induced studies whereas ours was resting-state. Third, previous innovation-connectivity patterns were identified in younger adults (ages 19–36 years), which may not necessarily be comparable to an older group (ages 56–75 years). Last, we were interested in neural changes following a 12-week CT protocol whereas many of the prior findings examined a single time point, with a few exceptions involving young adults (Fink et al., 2015; Saggar et al., 2016). We conclude that the functional changes in two neural networks relative to innovative cognition following training leaves a footprint in the resting state networks to better support enhanced innovative cognition in the aging brain.

This pilot study must be interpreted in the context of a number of limitations. First, the present task lacks the degree of validation of prior tasks used to measure divergent thinking, namely tasks which prompt for as many alternative uses of an object, (i.e., a “tissue”) as designed decades ago by Guilford (1967). Whereas we recognize this is a limitation and are in the process of establishing its validity; we propose that the task of deriving multiple interpretations for commonly encountered information may be a practical, functional task that is related to higher-order cognitive capacities that may have ecological validity. Other limitations include small sample size and lack of follow-up after training ended to shed light on the persistence of the gains. We were able to address whether this particular sample enhanced their performance from baseline. However, we were not able to evaluate whether individuals regained lost capacity or perhaps may be able to maintain and mitigate declining innovative cognition in the ensuing years. Addressing these latter issues requires longitudinal studies and quite possibly proactive interventions along the way to test whether declining abilities can be strengthened at life stages where decline emerges. Another possibility to consider for subsequent research is whether this older adult group achieved a level of performance that was superior to how they would have performed as a younger version of themselves. Some evidence suggests that the older mind may be able to take advantage of prior experience to engage in innovative cognition.

## Conclusion

The objective of the present study was to examine the effects of CT on innovative cognition in older adults. This study revealed that reasoning training via SMART improved innovative cognition which correlated to the key nodes of CEN and DMN networks. In sum, the current findings suggest that short-term and cost effective interventions, such as CT, may be beneficial in enhancing cognitive capacities and supporting neural mechanisms in healthy older adults. The new finding related to improved innovative cognition in healthy older adults is heartening; given innovative thinking is one of the most valued assets and fruitful outputs of the human mind across the lifespan (Kaufman and Sternberg, 2006; Palmiero et al., 2016). The potential to strengthen innovative cognition may tap into a positive and valuable resource of the aging mind that could support an individual’s ability to reinforce and retain an active mental lifestyle, engage in complex decision-making, intellect and psychological well-being with advancing age (Baltes et al., 1999; Kaufman and Sternberg, 2006). Much work needs to be done but this feasibility study motivates a continued push to harness the potential and reduce the gap of cognitive brain decline as we age.

## Author Contributions

SBC designed the study, interpreted the data and drafted the manuscript; JSS performed statistical analysis and drafted the manuscript; SA performed neuroimaging analysis, interpretation of neuroimaging data and reviewed the manuscript; MWK performed cognitive assessments and reviewed the manuscript.

## Conflict of Interest Statement

The authors declare that the research was conducted in the absence of any commercial or financial relationships that could be construed as a potential conflict of interest.

## Footnotes

^1^http://cran.r-project.org
