# Supplementary material for: Enhancing Innovation and Underlying Neural Mechanisms Via Cognitive Training in Healthy Older Adults
Source: Front Aging Neurosci. 2017 Oct 9;9:314. doi: 10.3389/fnagi.2017.00314 (PMC5640779; doi:10.3389/fnagi.2017.00314)
Supplement: Supplementary file 1 [file Image_1.pdf]

Figure S1. Flow diagram of the progress through the phases of a parallel randomized trial of 3 groups.

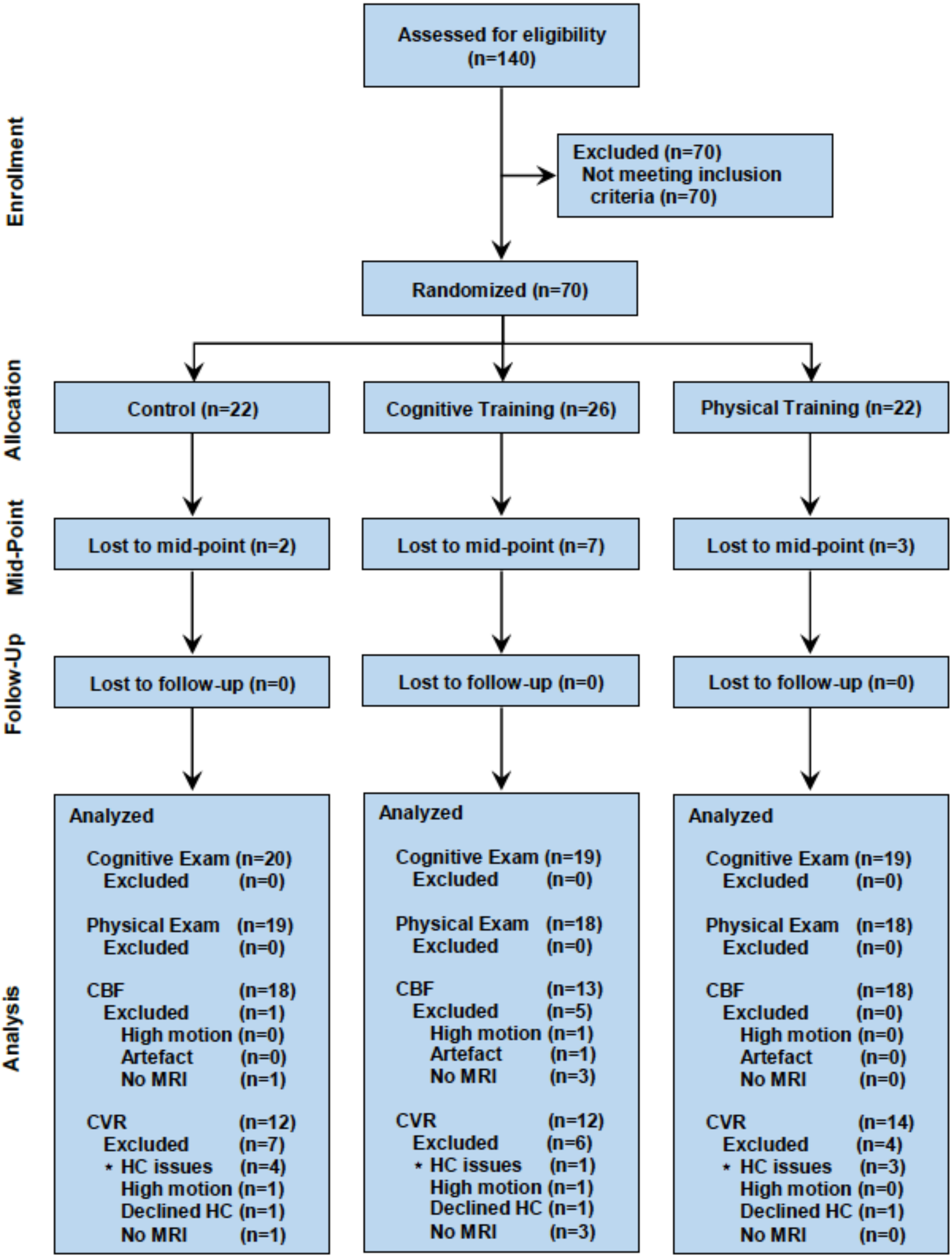

** HC Issues – Hypercapnia (HC) machine malfunctioned (e.g. no capnography recording), participant's nose clip fell off, subject hyper- or hypo-ventilated during the experiment.*
