# Supplementary material for: Enhancing Innovation and Underlying Neural Mechanisms Via Cognitive Training in Healthy Older Adults
Source: Front Aging Neurosci. 2017 Oct 9;9:314. doi: 10.3389/fnagi.2017.00314 (PMC5640779; doi:10.3389/fnagi.2017.00314)

Figure S2. QQ plot of studentized residuals for outlier diagnostics of the CEN model. One subject (lower left green circle) in the CT group was removed based on the studentized residual = -4.16, identified by outlier test (Bonferroni p-value =0.010).

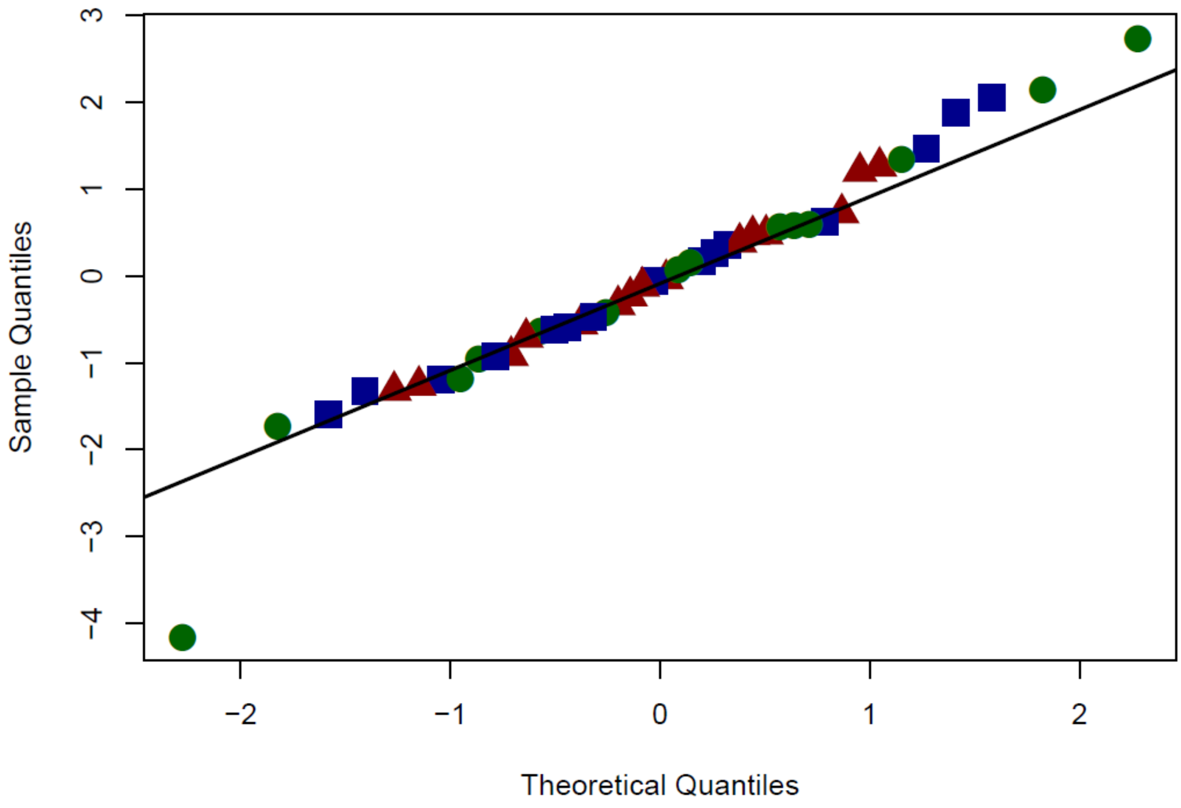

Supplement: Supplementary file 2 [file Image_2.pdf]
